# Social inequities in early childhood caries in Australia: A population‐based study on statewide public dental services data

**DOI:** 10.1111/ipd.13219

**Published:** 2024-05-30

**Authors:** Ankur Singh, Shalika Hegde, Mihiri Silva, Martin Whelan, Amalia Karahalios, David J. Manton, Sharon Goldfeld, Dallas R. English, Stuart Dashper

**Affiliations:** ^1^ Melbourne Dental School University of Melbourne Melbourne Victoria Australia; ^2^ Centre for Epidemiology and Biostatistics, Melbourne School of Population and Global Health University of Melbourne Melbourne Victoria Australia; ^3^ Dental Health Services Victoria Melbourne Victoria Australia; ^4^ Inflammatory Origins Murdoch Children's Research Institute Melbourne Victoria Australia; ^5^ Department of Dentistry Royal Children's Hospital Melbourne Victoria Australia; ^6^ Department of Paediatrics, Melbourne Medical School University of Melbourne Melbourne Victoria Australia; ^7^ Centrum voor Tandheelkunde en Mondzorgkunde University Medical Center Groningen Groningen The Netherlands; ^8^ Murdoch Children's Research Institute Melbourne Victoria Australia; ^9^ Cancer Epidemiology Division Cancer Council Victoria Melbourne Victoria Australia

**Keywords:** caries, child dentistry, dental public health, epidemiology, inequalities

## Abstract

**Background:**

Social disadvantage leads to dental caries during childhood.

**Aim:**

This study investigated whether dental caries occur earlier in children from households experiencing social disadvantage than those not experiencing social disadvantage.

**Design:**

The overall risk of, and relative time to, early childhood caries (ECC) according to sociodemographic characteristics in Victoria, Australia, was quantified. Records for 134 463 children in Victoria, Australia, from 2009 to 2019 were analysed. Time ratios (TR) and hazard ratios (HR) of carious lesion(s) in early childhood were estimated.

**Results:**

Compared with reference groups, Indigenous children had an adjusted TR of 0.80 (95% CI: 0.78, 0.82), children from households with languages other than English had an adjusted TR of 0.83 (95% CI: 0.82, 0.84), and dependants of concession cardholders had an adjusted TR of 0.81 (95% CI: 0.80, 0.81); therefore, 20%, 17% and 19% reduced times to the first carious lesion, respectively. The estimated HRs were 1.57 (95% CI: 1.49, 1.67) for Indigenous children, 1.46 (95% CI: 1.42, 1.50) for children from households with other languages and 1.57 (CI: 1.53, 1.60) for dependants of concession cardholders.

**Conclusion:**

Preventive oral health interventions must be targeted early in children from households experiencing social disadvantage to avoid social inequities in ECC.

**Why this paper is important to paediatric dentists**

- ECC occurs earlier among those experiencing social disadvantage than in children from advantaged backgrounds.
- There is an urgent need to target preventive oral health interventions and equitable oral healthcare delivery early to children and families experiencing social disadvantage.
- This study builds the evidence to integrate preventive oral health interventions into early childhood strategies to avoid social inequities in ECC.

## 1 INTRODUCTION

Health inequities are systematically generated unfair and unjust variations in health outcomes between population groups.^1^ The Lancet Commission on Oral Health has emphasised that the current approaches to maintaining oral health are ineffective in addressing inequities in oral health.^2^ Early childhood caries (ECC)* is defined as dental caries occurring in children younger than 6 years of age.^3^ International research has reported inequities in ECC. A recent umbrella review established low parental education, low family income and low social class as risk factors for ECC.^4^ In addition, associations between social disadvantage captured through household income, parental education, remoteness and ECC have been confirmed in many countries including Australia, Kuwait, Colombia, China, Thailand and the United States.^5^, ^6^, ^7^, ^8^, ^9^ The latest Australian National Child Oral Health Survey 2012‐14 reported that more than a quarter of five‐ to six‐year‐old Australian children had untreated ECC.^10^ One in three had already experienced ECC. Comparatively, 40% had untreated ECC, and half of all Indigenous children in Australia had experienced ECC.^10^

There is a gap in evidence regarding when ECC experience occurs, and whether the time to first ECC experience differs according to social disadvantage. Early and targeted preventive strategies according to social disadvantage for ECC are needed if children from households with social disadvantage experience dental caries earlier than those from households without social disadvantage.

ECC has disabling effects on children as it leads to problems with eating, speaking, educational achievement and socialisation.^11^, ^12^ When left untreated, ECC can cause pain and infection and may require invasive procedures under sedation or general anaesthesia, which are expensive, stretch health system resources and have negative impacts on children.^12^, ^13^ Addressing inequities in ECC is paramount if all children must have a solid foundation for good oral health regardless of their social or economic backgrounds. This study addresses critical gaps in the understanding of the origin of social inequities in ECC. This study aimed to quantify the risk of, and time to, ECC according to sociodemographic groups among children in Victoria, Australia.

## 2 MATERIALS AND METHODS

### 2.1 Data source and participants

We analysed statewide routinely collected data of 186 489 records obtained from 144 938 children below 6 years of age between 2009 and 2019 from the public dental health services (Dental Health Services Victoria; DHSV), Victoria, Australia. Children under 6 years of age are eligible for public dental care in Victoria. DHSV maintains data on clinical diagnoses, treatment history, services provided and demographic characteristics (age, sex, Indigenous status, language spoken at home, parent concession cardholder status [those receiving government benefits]) and postcode using Titanium® (Titanium Solutions Ltd, Auckland, New Zealand), an electronic patient management system. In Australia, concession cards are allotted to someone who receives a specific government payment, pension or allowance (age pension, carers pension, disability allowance, disability support pension, family allowance, family payment, JobSeeker payment, low‐income allowance, mobility allowance, Newstart, partner allowance, parenting payment [single or partnered], parenting/partner allowance, sickness allowance, special benefit, widow allowance, wife pension and widow pension). The data are collected to analyse and inform the planning of dental health services. Of the 144 938 children, data were used from 134 463 children (93%) with no missing data and accurate records. For some children, the date of the first dental visit preceded the date of birth, likely due to data entry errors, and they were excluded from the analysis. A flow chart of participant selection is shown in Figure S1.

This observational study conformed to the Strengthening the Reporting of Observational Studies in Epidemiology guidelines.

### 2.2 Exposure measures

Four separate exposures were examined. First, we examined Indigenous status. At the time of registration, participants (parents/guardians) self‐identify as Aboriginal or Torres Strait Islander or non‐Indigenous. Second, we considered the main language spoken at home. We categorised this variable as a binary variable (English/other). Third, we examined households provided with government benefits and a concession card as they experience a substantial disadvantage in Australia. Children from such households were recorded as dependants of concession cardholders (henceforth, cardholders for brevity) and those without were non‐cardholders. Our final exposure was a measure of area deprivation. Participants' postcodes were matched to the area‐based Index for Relative Socioeconomic Disadvantage (IRSD) provided by the Australian Bureau of Statistics. IRSD scores are part of the Australian Socio‐Economic Indexes for Areas (SEIFA), which are composite scores for deprivation generated at a small area level. Tertiles for IRSD scores were created to compare the incidence of carious lesions among children: Tertile 1 corresponding to lowest disadvantage and Tertile 3 to highest disadvantage. Data on each of these variables were collected at the first contact with the public dental health services, and information on cardholder status was updated if the status of parents/guardians changed at subsequent visits. For statistical analysis, we used the baseline measures of covariates—data recorded at the first contact.

### 2.3 Outcome measures

As part of each clinical examination, dental professionals identify and record cavitated carious lesions on a tooth chart using the Titanium®^−^ software. A total decayed, missing, filled teeth (dmft) score is then automatically computed by the software and recorded for each patient. A dmft score of one or more indicates experience of carious lesion, whereas a score of zero indicates no experience of carious lesion.

### 2.4 Covariates

We included age at first clinic visit, derived from date of birth and date of the first dental visit, year of first clinic visit (2009–2019), sex of the child (male, female) and geographic remoteness (metropolitan, inner regional, outer regional, remote and very remote)^14^ as covariates.

### 2.5 Statistical analysis

The exact timing of cavitation was unknown, and only the timing of the dental visits between when it occurred was known; thus, an interval‐censored survival analysis method was adopted (Figure S2). We quantified the risk of developing carious lesions in relation to each of the exposure measures by fitting separate multivariable interval‐censored parametric Weibull regression models with age as the time metric, allowing accelerated failure time models and a proportional hazards model while accommodating for interval censoring. This approach acknowledges the irregular time intervals between dental visits, differences in dental visit participation rates and the period over which dental visits occur.^15^ A study participant was left‐censored if ECC was present at the first visit and right‐censored if carious lesions were not present until their last visit prior to the age of 6. We used the date of birth recorded within the dental health records as time zero and subtracted each visit date from date of birth to estimate the follow‐up duration. We estimated two measures from each multivariable model—the adjusted hazard ratio (HR), which is the incidence rate of developing ECC among exposed compared with the unexposed; and time ratios (TR) from accelerated failure time models that are interpreted as the ratio of median time to event for exposed compared with unexposed. We plotted the age‐specific risks (cumulative incidence) of developing carious lesions from multivariable models by exposure categories that indicate the proportion of children who have lesions. All models accounted for confounding factors as described below.

For each multivariable model, the children experiencing advantage were considered as the reference groups (i.e., non‐Indigenous children, English‐speaking households and non‐cardholders). When Indigenous status and language spoken at home were the exposures, we adjusted for age, year of first dental visit, child sex, geographic remoteness (major cities, inner regional and outer regional/ remote/ very remote areas), area deprivation and cardholder status. When cardholder status was the exposure, we additionally adjusted for Indigenous status and language spoken at home, and when area deprivation was the exposure, we adjusted for Indigenous status and language spoken at home and cardholder status. Confounding adjustment was carried out as per the disjunctive cause criterion.^16^ All analyses were conducted using Stata 16 (StataCorp, College Station, TX, USA).

## 3 RESULTS

The first dental visit for most children was at ages four or five years (*n* = 83 144, 62%). Two per cent (*n* = 2841) of the children were Indigenous, 12% (*n* = 15 795) were from households with languages other than English, and 44% (*n* = 58 455) of the children were cardholders. There was an equal distribution of male and female children. Most were from major cities (66%) (Table 1).

**TABLE 1:** Descriptive characteristics of the sample on the first visit (Time(0)) (*n* = 134 463).

Characteristic	Category	Number (%)/median (25th/75th percentile)
Age at first visit (years)	1	5993 (4.5)
	2	16 039 (11.9)
	3	29 287 (21.8)
	4	41 527 (30.9)
	5	41 617 (31.0)
Sex	Male	67 135 (50.0)
	Female	67 328 (50.0)
Indigenous status	No	131 619 (97.9)
	Yes	2844 (2.1)
Language spoken at home	English	118 668 (88.3)
	Others	15 795 (11.8)
Cardholder	No	75 825 (56.4)
	Yes	58 638 (43.6)
Remoteness	Major Cities	89 319 (66.4)
	Inner regional	37 682 (28.0)
	Outer regional/remote	7462 (5.6)
Number of follow‐up visits after Time(0) (Median (25th/75th percentile))		1 (1, 1)
Days from caries‐free visit to first visit with diagnosis of carious lesion (*n* = 9088) (Median (25th/75th percentile))		439 (343, 661)

Of the 134 463 children, 40 145 children (30%) had a dmft score > 0 at the first appointment and 5870 (4%) children developed ECC by the age of six (Figure S3).

The adjusted HR showed that children in each exposed category had a higher risk of experiencing carious lesions than those in the reference category (Table 2). The HR of experiencing carious lesions at any time during the follow‐up to 6 years compared with their respective reference categories were as follows: Indigenous children, 1.57 (95% CI: 1.49, 1.67); children from households with language other than English, 1.46 (95% CI: 1.42, 1.50); cardholders, 1.57 (95% CI: 1.53, 1.60); and those from deprived areas, 1.22 (95% CI: 1.19, 1.25) for second tertile and 1.45 (95% CI: 1.41, 1.48) for third tertile (Table 2).

**TABLE 2:** Individual measures of social position and presence of dental caries (*n* = 134 463).

Category	Children experiencing carious lesion (*n*)	Total (*n*)	Adjusted hazard ratio (95% CI)	Adjusted time ratio (95% CI)
Indigenous status				
Non‐Indigenous Australians	44 715	131 619	1^a^	1^a^
Indigenous Australians	1300	2844	1.57 (1.49, 1.67)	0.80 (0.78, 0.82)
Language spoken at home				
English	38 922	118 668	1^a^	1^a^
Others	7093	15 795	1.46 (1.42, 1.50)	0.83 (0.82, 0.84)
Cardholder status				
No	20 424	75 825	1^b^	1^b^
Yes	25 591	58 638	1.57 (1.53, 1.60)	0.81 (0.80, 0.81)
Area level of deprivation				
First tertile (least disadvantaged)	13 146	48 507	1^c^	1^c^
Middle tertile	12 618	36 997	1.22 (1.19, 1.25)	0.91 (0.90, 0.92)
Highest tertile (most disadvantaged)	20 251	48 959	1.45 (1.41, 1.48)	0.84 (0.83, 0.85)

^a^ Adjusted for age of first visit, sex, year of first visit, area of remoteness and area level of deprivation.

^b^ Adjusted for age of first visit, sex, Indigenous status, preferred language, year of first visit, area of remoteness and area level of deprivation.

^c^ Adjusted for age of first visit, sex, Indigenous status, preferred language, year of first visit and cardholder status.

**FIGURE 1:**
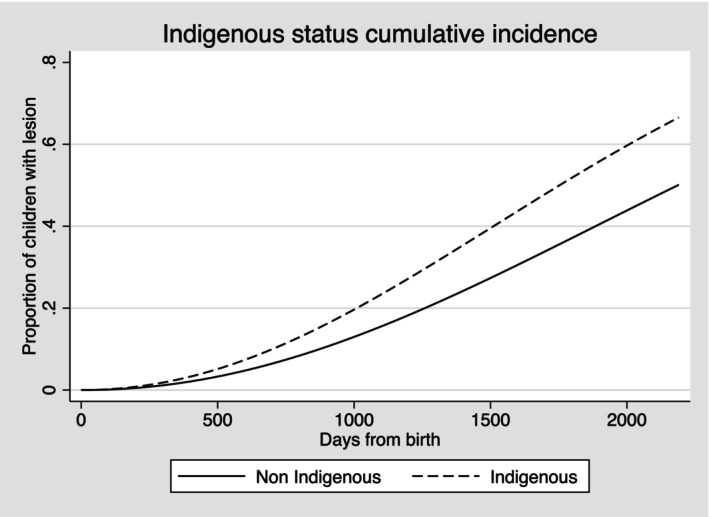
Cumulative incidence of carious lesions by Indigenous status.

**FIGURE 2:**
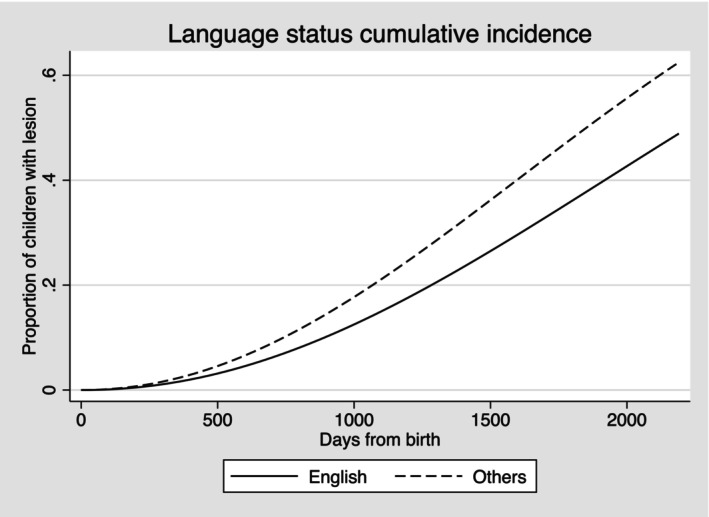
Cumulative incidence of carious lesions by language spoken at home status.

**FIGURE 3:**
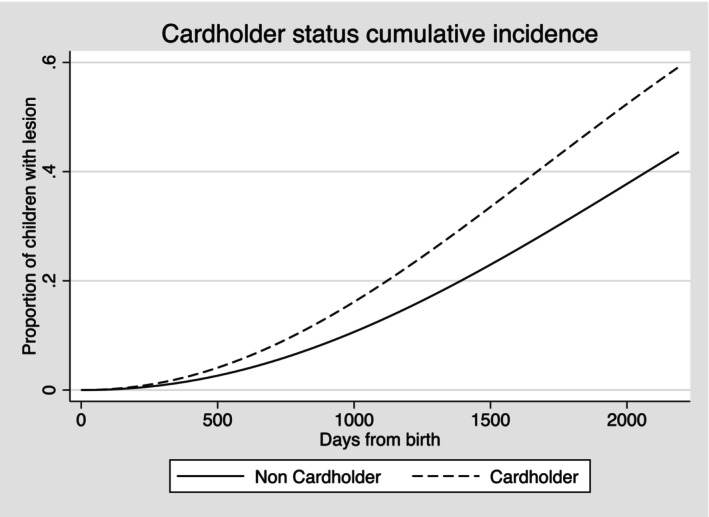
Cumulative incidence of carious lesions by concession cardholder status.

The plots for cumulative incidence showed the overall proportion of lesion‐free children who developed carious lesions by the end of the follow‐up period. Six in 10 children who were Indigenous, or from households with language other than English, experienced a carious lesion when compared to 4 in 10 non‐Indigenous children or from English‐language households. Similar differences were seen also in risk for cardholders compared with non‐cardholders (Figures 1, 2, 3). A similar pattern was also confirmed for area deprivation (Figure S3).

The adjusted TRs for failure (experience of carious lesion) showed 20% (95% CI: 18%, 22%) shorter time to event for Indigenous children, 17% (95% CI: 16%, 18%) shorter for children from households with languages other than English, 19% (95% CI: 19%, 20%) shorter for children who were dependants of concession cardholders and 16% shorter (95% CI: 15%, 17%) for children from the most deprived areas compared with the reference groups.

## 4 DISCUSSION

We aimed to determine the risk and timing of carious lesion development according to the sociodemographic background of pre‐school children using a large clinical database of children attending public dental services in Victoria, Australia. Our findings showed that carious lesions develop earlier in children from households or sociodemographic backgrounds with relatively higher social disadvantage than those from advantaged backgrounds. Our findings were consistent across four separate measures of sociodemographic backgrounds (Indigenous status, language spoken at home, concession cardholder status and area deprivation).

The key strength of our analysis is that we were able to examine differences in carious lesions in Indigenous children and children from culturally and linguistically diverse backgrounds who usually lack representation in existing birth cohort studies, particularly examining oral health outcomes, a well‐known limitation in this area of research.^13^ Most surveys of child oral health enrol school children at the age of 5 or 6 years,^10^ providing little information on the prevalence of ECC at the population level in the period prior to 5 years of age, a time when risk factors of ECC are already established. In addition, our study exploits an interval‐censored survival analysis technique on a large routinely collected data set to quantify the timing of carious lesions by different sociodemographic backgrounds.

Our study has some limitations. Children in Victoria have access to both public and private dental health care, and the public dental system mainly serves the population experiencing disadvantage for whom private dental health care is unaffordable. There is potential for selection bias if accessing public dental care is a common outcome of both social disadvantage and having carious lesions. The advantage of using interval‐censored survival analysis is that it left censors children who have carious lesions at their first dental visit. Another limitation of our study is the potential for misclassification of the outcome due to the administrative nature of the data and exposures. This is, however, unlikely to be differential as there is no reason for the dental workforce to differentially report disease outcomes according to the social characteristics we assessed. Our findings are only generalisable to children attending public dental services in Victoria. In addition, data are obtained through separate procedures—clinical examination and self‐reported forms. Finally, most children in our study attended public dental services around 4 years of age, which is close to the end for ECC. Therefore, we could not fully examine social inequities in ECC at earlier ages coinciding with tooth eruption. Additionally, due to this skewed data absolute measures (median time) quantifying time to event can be misleading, and therefore, we only present relative measures for time to event (TRs). It must be noted that the period of risk for ECC is short, from teeth eruption to 6 years of age. We did not examine the severity of ECC as the outcome nor interactions between different measures of social disadvantage. These must be considered as important research implications and examined in future research.

Social inequities in dental caries in children are reported internationally and in Australia.^2^, ^5^, ^6^, ^7^, ^8^, ^12^, ^17^ It is known that social inequities in oral health outcomes are likely to arise due to impacts of social determinants of health.^17^ Yet, evidence is lacking on the nature and extent of social inequalities in ECC due to the lack of population‐based data. The present study fills this important gap in research. Current studies that have focussed on the time of carious lesion development as an outcome have found levels of salivary mutans streptococci and previous caries experience as risk factors for caries and reported protective effects of drinking fluoridated tap water in reducing caries incidence.^18^, ^19^ The main contribution of our paper is finding an increased risk of ECC for children experiencing social disadvantage throughout the childhood period and that the time of occurrence of ECC is earlier in children experiencing social disadvantage than in the advantaged backgrounds. The presence of a cavitated carious lesion, which is an advanced stage of lesion development, suggests the need for earlier targeting of intervention(s) to reduce ECC among children experiencing social disadvantage.

There are important policy implications from our findings. Settings such as childcare centres must be targeted for oral health promotion interventions, particularly those serving children from social and demographic backgrounds at a higher risk of ECC. Our findings suggest that interventions must be evaluated rigorously for their equity impacts in early life. For instance, there is supporting evidence that community water fluoridation is an effective population‐level intervention to reduce inequalities in the caries experience of children.^20^ All well‐intended public health interventions do not reduce inequalities in health outcomes, and ‘downstream’ interventions (those targeting knowledge and attitudes) may increase inequalities in ECC.^21^ In addition, interventions of health education are known to have little effect on ECC and on sugar consumption (the essential cause of ECC).^22^, ^23^ We analysed data on children accessing dental services. We postulate that a range of interventions in addition to access to dental health care—fluoride, nutritional environment, social and economic interventions—should be considered in preventing the inequalities in ECC.^20^ Finally, health systems need to ensure the provision of appropriate clinical services, including specialised services to restore health in these children. Although preventing the incidence of dental caries is pivotal, our findings indicate that equitable access to treatments is also key to reduce inequalities in severe consequences of dental caries in children.

Social inequalities in dental caries have an early onset. Preventive oral health interventions and oral healthcare services must be targeted early and equitably delivered to sociodemographic populations to avoid social inequalities in ECC. A need exists for precision strategies for preventing ECC, particularly among children with dissociodemographic backgrounds at a higher risk of ECC.

## AUTHOR CONTRIBUTIONS

AS, SH, MW, DJM and SD conceptualised and designed the study. SH and MW contributed to the acquisition of data. AS, AK and DRE contributed to the analysis and interpretation of the data. AS drafted the article, and SH, SH, MW, DJM, SD, AK, DRE, MH and SG revised it critically for important intellectual content. All authors approved the final manuscript as submitted, and agreed to be accountable for all aspects of the work. All authors have no conflict of interest to declare.

## FUNDING INFORMATION

Ankur Singh is funded by the Australian Research Council DECRA Fellowship (DE230101210). Mihiri Silva is funded by a Melbourne Children's Clinician‐Scientist Fellowship. Sharon Goldfeld is supported by the NHMRC Practitioner Fellowship (1155290).

## CONFLICT OF INTEREST STATEMENT

No conflicts of interest to declare.

## Supporting information


Appendix S1

## Footnotes

* Early childhood caries (ECC); Dental Health Services Victoria (DHSV); Index for Relative Socioeconomic Disadvantage (IRSD); Australian Socio‐Economic Indexes for Areas (SEIFA).
