# Supplementary material for: Social inequities in early childhood caries in Australia: A population‐based study on statewide public dental services data
Source: Int J Paediatr Dent. 2024 May 30;35(1):186–93. doi: 10.1111/ipd.13219 (PMC11626491; doi:10.1111/ipd.13219)
Supplement: Supplementary file 1 — Appendix S1 [file IPD-35-186-s001.docx]

Supplementary materials

# Flowchart of sample


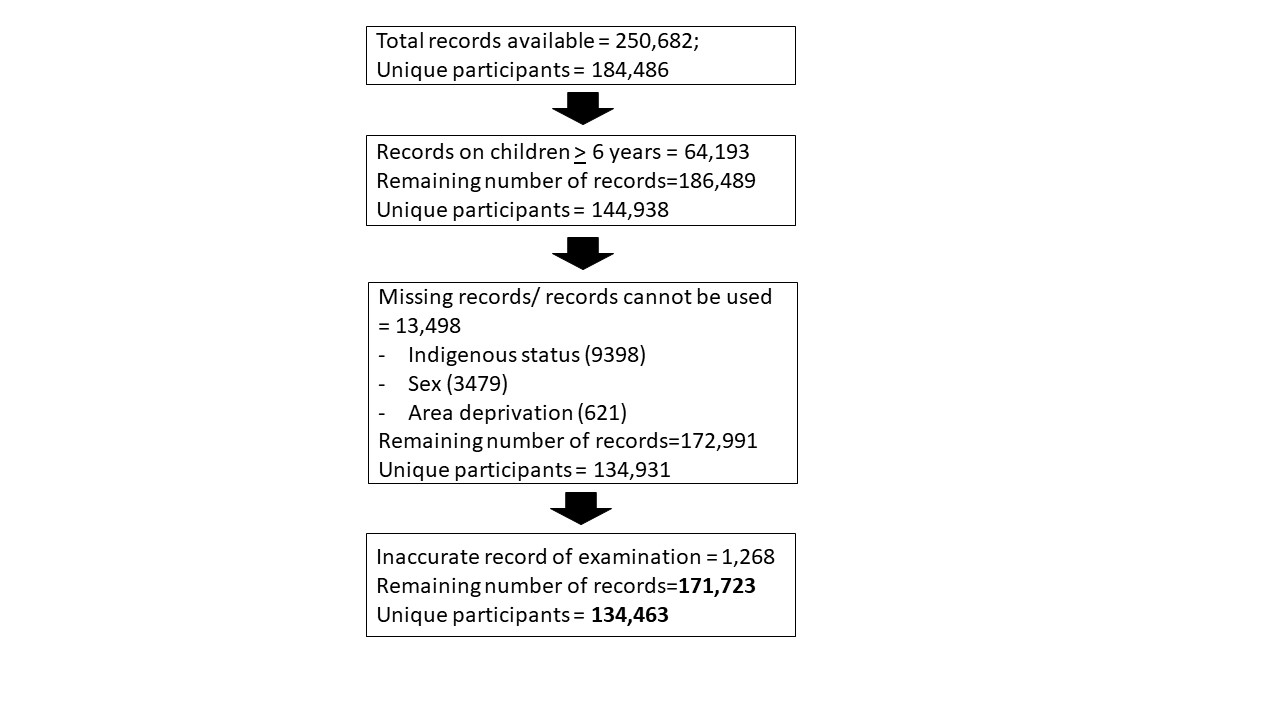


Supplementary Figure 1 Flowchart of analytical sample.

# Differences in background characteristics by missing data status

|  |  | Missing data |  | P-value |
| --- | --- | --- | --- | --- |
|  |  | No | Yes |  |
| Dental caries | No | 92.8 (91,415) | 7.2 (7,119) | 0.453 |
|  | Yes | 92.9 (43,102) | 7.1 (3,302) |  |
| Indigenous status | No | 97.6 (131,645) | 2.4 (3,235) | 0.003 |
|  | Yes | 96.8 (2,872) | 3.2 (96) |  |
| Cardholder status | No | 93.3 (75,809) | 6.8 (5,490) | <0.001 |
|  | Yes | 92.3 (58,708) | 7.8 (4,931) |  |
| Sex | Male | 94.7 (67,160) | 5.3 (3,772) | 0.18 |
|  | Female | 94.8 (67,357) | 5.2 (3,664) |  |
| AgeInt | 1 | 91.5 (4,805) | 8.5 (447) | 0.005 |
|  | 2 | 92.7 (12,896) | 7.3 (1,010) |  |
|  | 3 | 92.8 (25,387) | 7.2 (1,974) |  |
|  | 4 | 92.9 (41,974) | 7.1 (3,217) |  |
|  | 5 | 92.9 (49,455) | 7.1 (3,773) |  |
| Language spoken at home | English | 93.9 (118,952) | 6.1 (7,722) | <0.001 |
|  | Others | 85.2 (15,565) | 14.8 (2,699) | 0.003 |
| Area deprivation | Low | 93.4 (48,626) | 6.6 (3,429) |  |
|  | Medium | 93.1 (37,033) | 6.9 (2,747) |  |
|  | High | 92.9 (48,858) | 7.1 (3,741) |  |

# Censoring


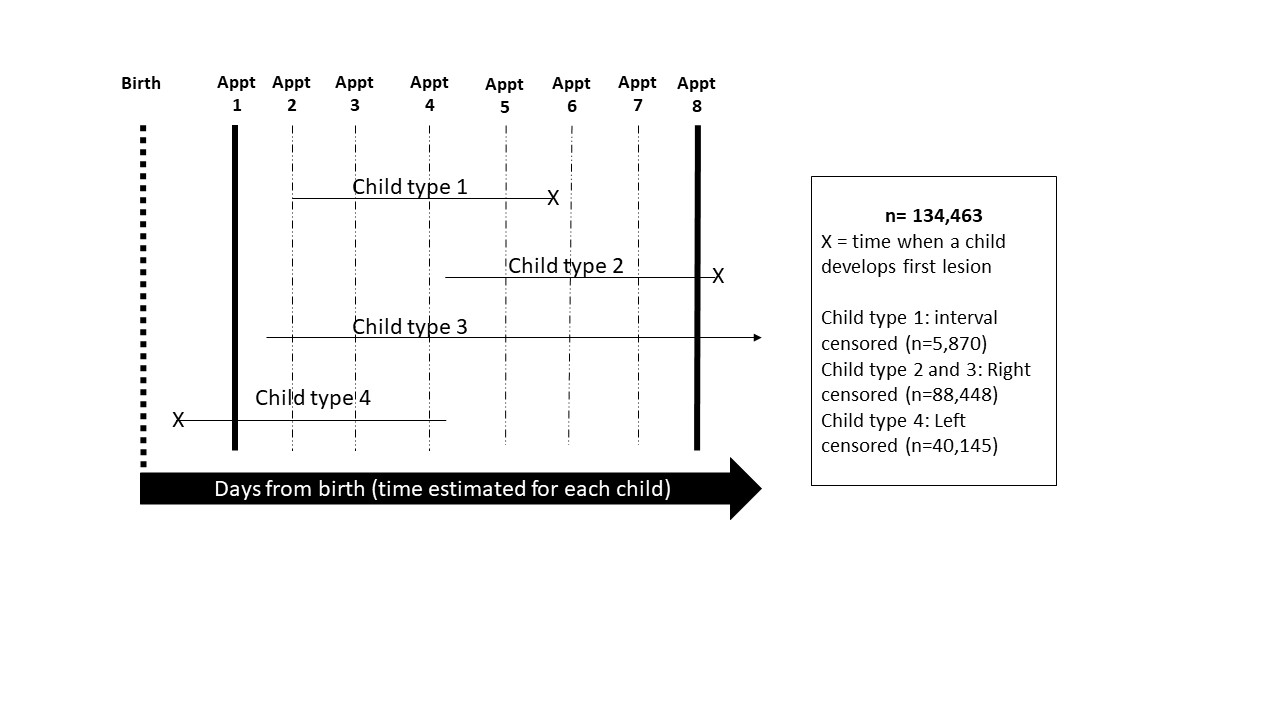


Supplementary Figure 2. Schematic figure showing left, right and interval censoring as per case status in the analysed sample (n=134,463)

# Deprivation


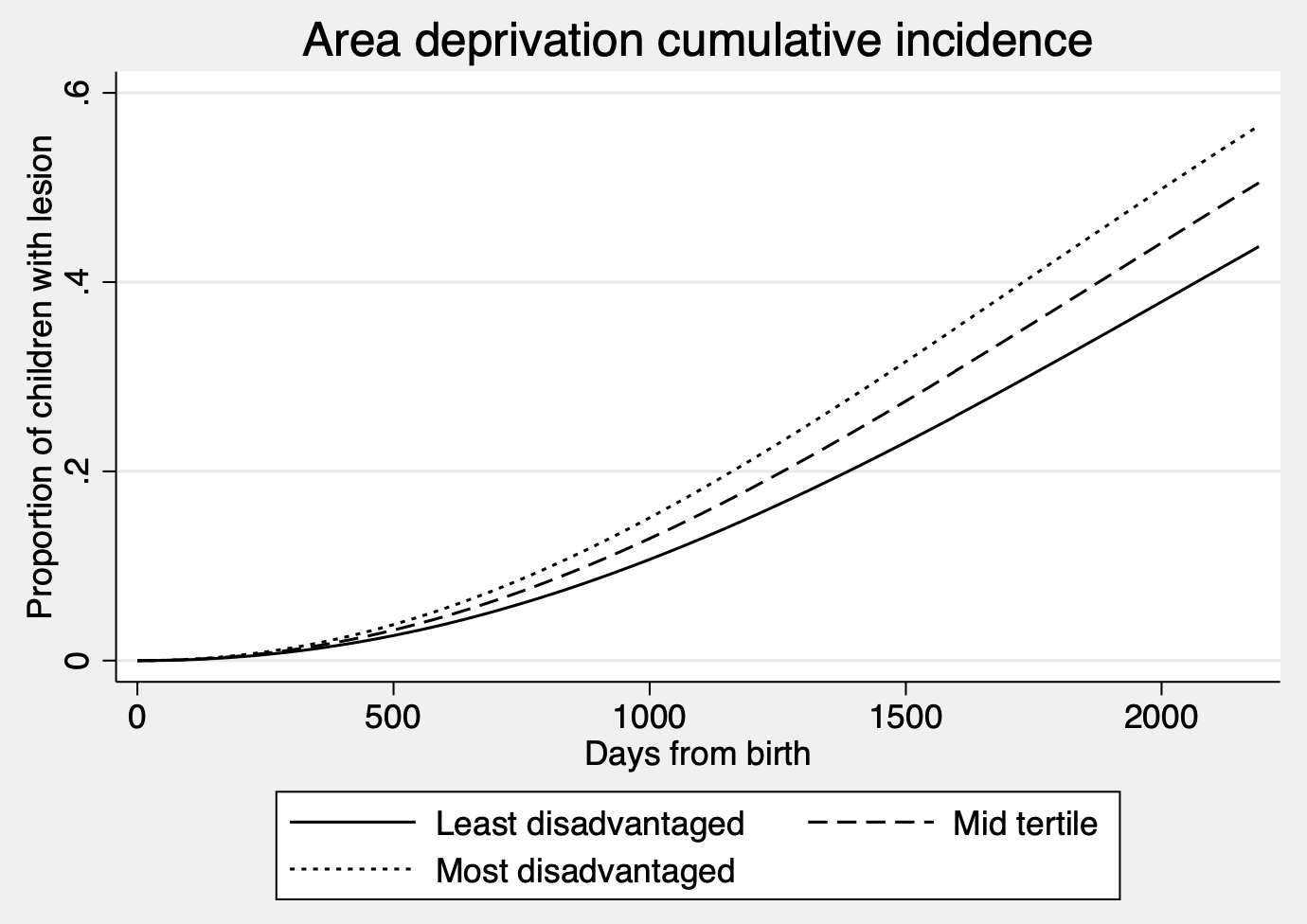


Supplementary Figure 3. Figure showing cumulative incidence of carious lesions by area deprivation
